# Supplementary material for: microRNA-mRNA Analysis Reveals Tissue-Specific Regulation of microRNA in Mangrove Clam (Geloina erosa)
Source: Biology (Basel). 2023 Dec 11;12(12):1510. doi: 10.3390/biology12121510 (PMC10740791; doi:10.3390/biology12121510)
Supplement: Supplementary file 1 [file biology-12-01510-s001.zip › Supplementary Annotation.docx]

**Supplementary Table S1**

**Differential Expression and Enrichment Analysis**

2-1 gill_vs_ctrl

The results of the differential expression analysis between gills and controls (hepatopancreas, muscles)

2-2 g_vs_ctrl_enrichment

The enrichment analysis results of differentially expressed miRNAs between gills and controls (hepatopancreas, muscles)

2-3 hepa_vs_ctrl

The results of the differential expression analysis between hepatopancreas and controls (gills, muscles)

2-4 hepa_vs_ctrl_enrichment

The enrichment analysis results of differentially expressed miRNAs between hepatopancreas and controls (gills, muscles)

2-5 musle_vs_ctrl

The results of the differential expression analysis between muscles and controls (gills, hepatopancreas)

2-6 musle_vs_ctrl_enrichment

The enrichment analysis results of differentially expressed miRNAs between muscles and controls (gills, hepatopancreas)

**For differential expression (2-1, 2-3, 2-5)**：

These columns provide important information about the gene expression changes and their statistical significance in the DESeq2 analysis.

baseMean: It represents the average count of reads for a given gene across all samples in the experiment. It is a measure of the overall expression level of the gene.

log2FoldChange: It indicates the log2-fold change in gene expression between two conditions or groups. It is calculated as the difference in expression levels between the two conditions on a logarithmic scale.

lfcSE: It stands for log2-fold change standard error. It represents the standard error associated with the log2-fold change estimate and provides information about the uncertainty or variability of the fold change calculation.

stat: It refers to the statistical test statistic value, which is used to assess the significance of the log2-fold change. It is typically based on a statistical test such as a Wald test or likelihood ratio test.

pvalue: It represents the p-value associated with the statistical test. The p-value indicates the probability of observing the data under the null hypothesis, assuming there is no true difference in gene expression between the conditions.

padj: It stands for adjusted p-value, which takes into account multiple testing corrections to control the false discovery rate (FDR). It is often calculated using methods such as the Benjamini-Hochberg procedure.

**For enrichment analysis(2-2, 2-4, 2-6)**

BP: biological process.

CC: cellular component.

MF: molecular function.

KEGG: Kyoto Encyclopedia of Genes and Genomes.

KOG: EuKaryotic Orthologous Groups (KOG).

PFAM: protein family.

**Supplementary Table S2**

**miRNA co-expression analysis results by WGCNA.**

3-1 module_trait_cor

Table of related items for modules and traits (tissue types). Corresponds to Figure 8 (A).

3-2 module_miRNAs

Correspondence between miRNA and module (color).

3-3 gill

Enrichment analysis results of gill related MEblue module.

3-4 hepa

Enrichment analysis results of MEblack module related to hepatopancreas.

3-5 muscle

Enrichment analysis results of muscle related MEpurple module.

**For enrichment analysis (3-3, 3-4, 3-5):**

GO_GO_BP: In Gene Ontology, the enrichment analysis results of BP: biological process.

GO_GO_CC: Enrichment analysis results of cellular component (CC) in gene ontology.

GO_GO_MF: In gene ontology, the enrichment analysis results of MF: molecular function.

KEGG: Kyoto Encyclopedia of Genes and Genomes.

KOG: EuKaryotic Orthologous Groups (KOG)

PFAM: protein family

**Supplementary Table S3**

**miRNA Expression Profile**

**Supplementary Data Sequence**

**miRNA Sequences**

This file provides the sequences of all miRNAs. Each row represents a gene, and each column represents a sample.

All_miRNA.expressed:

Sequences of all expressed miRNAs.

Known_miRNA.expressed

Sequences of expressed miRNAs predicted to be homologous to known miRNAs.

Novel_miRNA.expressed

Sequences of expressed miRNAs predicted to be novel.
